# Mediating role of social capital on the association between negative life events and quality of life among adults in China: A population-based study

**DOI:** 10.3389/fpubh.2022.987579

**Published:** 2022-09-29

**Authors:** Jianghui Zhang, Songmei Wang, Xuehui Zhang, Xiaoyu Han, Haoyuan Deng, Nan Cheng, Yunrui Sun, Chongwei Song, Zhongxin Hou, Jianzhong Yin, Qiong Meng

**Affiliations:** ^1^School of Public Health, Kunming Medical University, Kunming, China; ^2^Yunnan Provincial Center for Disease Control and Prevention, Kunming, China; ^3^Baoshan College of Traditional Chinese Medicine, Baoshan, China

**Keywords:** negative life events, social capital, quality of life, structural equation modeling, mediation effect

## Abstract

**Objective:**

To evaluate whether social capital played a mediating role in the relationship between negative life events (NLE) and quality of life (QoL) among adults in China after proposed a conceptual model based on stress buffering theory.

**Methods:**

A cross-sectional study was conducted based on baseline survey from the Chinese Multi-ethnic Cohort (CMEC) in Yunnan province. A total of 22,866 adults were recruited by multistage stratified cluster sampling. A structured questionnaire was used to collect the general demographic characteristics and the occurrence of NLE. A self-developed brief social capital scale was utilized to assess the social capital and the EQ-5D-5L scale was used to measure the QoL. The relationships among NLE, social capital and QoL were analyzed using multiple linear regression analyses. Structural equation models were used to evaluate whether social capital had a mediating effect on the relationship between NLE and QoL. The standardize coefficient (β) and it's 95% confidence intervals (CI) were calculated in this process.

**Results:**

The mean age of participants was 52.70 years old. The mean of EQ-5D index value was 0.92 (SD = 0.12) and the mean of EQ-VAS score was 71.77 (SD = 13.80). NLE not only directly affected EQ-5D index value [β = −0.127, 95% CI (−0.144, −0.110)] but also indirectly negatively affected EQ-5D index value through social capital [β = −0.019, 95% CI (−0.023, −0.015)]. Social capital had a direct positive effect on EQ-5D index value [β = 0.114, 95% CI (0.097, 0.133)]. A similar pattern was identified for the association between NLE and EQ-VAS score. The direct effect of NLE on EQ-VAS score was negatively significant [β = −0.132, 95% CI (−0.146, −0.118)]. Moreover, the indirect effect through social capital was also negatively significant [β = −0.022, 95% CI (−0.026, −0.019)]. There was a positive direct effect of social capital on EQ-VAS score [β = 0.135, 95% CI (0.117, 0.151)].

**Conclusion:**

Social capital played an important mediating role in the relationship between NLE and QoL, and it alleviated the negative effects of NLE on the QoL of the community residents in China. Providing reliable social capital for community residents experiencing NLE could effectively improve their quality of life.

## Introduction

Good health and well-being are an important part of the United Nations Sustainable Development Goals (SDGs), and good health can contribute to the achievement of other SDGs. Therefore, quality of life (QoL) research has attracted extensive attention from domestic and foreign researchers (1–3). QoL is a multidimensional concept, defined by the World Health Organization as the perception of individuals in different cultural contexts and value systems of their living conditions in relation to their goals, expectations, standards, and concerns (4); it reflects the overall well-being in terms of an individual's physical and mental health. QoL is affected by many factors, which can be roughly divided into two categories: the internal factors of the individual, such as gender, age (5), race (6), and personality characteristics (7, 8), and external factors, such as negative life events (9) and social support (10).

Social capital plays an important role in maintaining physical health as a vital human resource. Rostila (11) defines social capital as “Social capital comprises the social resources that evolve in accessible social networks or social structures characterized by mutual trust. These social resources, in turn, facilitate access to various instrumental and expressive returns, which might benefit both the individual and the collective.” Social networks, social support, trust and participation are widely considered to be the key elements of social capital. Many studies have shown a significant correlation between QoL and social capital. For example, Zhong (12) suggested that increased social capital could improve the QoL of elderly individuals in rural China. RezaeiNiar (13) found that social capital has a significant positive association on the physical and mental health dimensions of QoL for pregnant women. Some longitudinal analysis showed that social capital had significant effect on total mortality rate and depressive symptoms (14, 15). However, studies on the correlation between social capital and QoL have mostly focused on the elderly population and patients, while there is a lack of relevant studies of the community adults.

Life events refer to a variety of social life changes that people encounter in their daily lives. According to their impact on individual health, life events can be divided into negative life events (NLE) and positive life events (PLE). NLE are recognized as a psychosocial stress factor, and with the transformation of the biological-psychosocial-social medicine model, an increasing number of scholars have paid attention to the influence of NLE on individual physical and mental health in recent years. Life events can increase the risk of depression (16, 17), affect individual mental health (18), and reduce one's subjective well-being (19). In recent years, some studies (2, 10, 20) have shown that NLE are significantly related to QoL.

QoL is a multidimensional index that includes physical health, mental health and social function. Although NLE are stressful for individuals, the QoL of individuals who experience NLE does not necessarily decrease. How individuals respond to NLE is influenced by many factors. Some of these factors (such as social capital) may act as a buffer against the loss of an individual's QoL. According to the buffer theory by Cohen and Wills (21), social resources can alleviate the impact of stressful life events on individual health and protect individuals from stress damage. Under the stress buffering model, social support was considered as an important variable that weakens the impact of NLE on health. Social support may act on different points in the pathway between NLE and illness. First, social support can intervene between NLE and stress responses by assessing life events and weakening stressful responses (21, 22). The availability of social source in the face of negative life event may lead to reassess the potential harm caused by stress, or improve the individual's ability to cope with demands, thereby reducing the individual sees a specific life events as the possibility of high pressure. Second, appropriate support can intervene in the occurrence of stress subjective experiences and behavioral patterns by reducing or eliminating stress responses or directly affecting physiological processes. Previous studies have shown that social support plays a positive role in relieving the negative impact of NLE on adolescent depression (23) and that it can reduce the negative effects of life events on individuals' mental health (24). A study among People Living with HIV/AIDS (PLHIV) in China showed that social support played an important moderating role in the relationship between traumatic life events and QoL (25). Social capital can provide individuals with connections to receive social support from network members when needed, so social support can be seen as an outcome of social capital (26). Although the buffering effect of social capital has been discussed in other studies, no studies have examined the effect of social capital between NLE and QoL in the community adults. Therefore, the purpose of this study was to evaluate whether social capital played a mediating role in the relationship between negative life events and quality of life (QoL) among the community adults in China by using structural equation modeling to analyze the measurement data about NLE, social capital and QoL from the baseline survey of the Yunnan Cohort in the Chinese Multi-ethnic Cohort.

## Materials and methods

### Study design and participants

A cross-sectional study was conducted based on baseline survey from the Chinese Multi-ethnic Cohort (CMEC) in Yunnan province, which was conducted from May 2018 to September 2019. The CMEC has been described in great details in the previous research (27). Briefly, the CMEC was established in five provinces (Chongqing, Guizhou, Sichuan, Tibet and Yunnan) in Southwest China. The sample in this study was obtained from community-based populations in Yunnan province using a multistage stratified cluster sampling method. In the first stage, four minority settlements (Yongsheng County, Heqing County, Yongren County and Wuding County) were selected as our study sites. In Yongsheng County, the total population is 336,832, and Han ethnic group accounts for 65.07%. The total population of Heqing County is 243,031, and the Bai ethnic group accounts for 59% of the total population. The total population of Wuding County is 236,500, with the Yi ethnic group accounting for 32.08%. The Yi ethnic group of Yongren County is 58,573, accounting for 55.5% of the total population. In the second stage, two to eight communities (depending on the size of communities) in each settlement were selected considering the migration status, local health conditions. In the final stage, all participants met our inclusion criteria were invited to take part in our study in consideration of both sex ratio and age ratio. The inclusion criteria used to recruit study participants were below: (i) aged 30–79 years on the day of investigation; (ii) was permanent residents and available to complete baseline survey and will be available to complete follow-up studies; and (iii) was able to complete the questionnaire interview, physical examination and blood tests.

**Figure 1:**
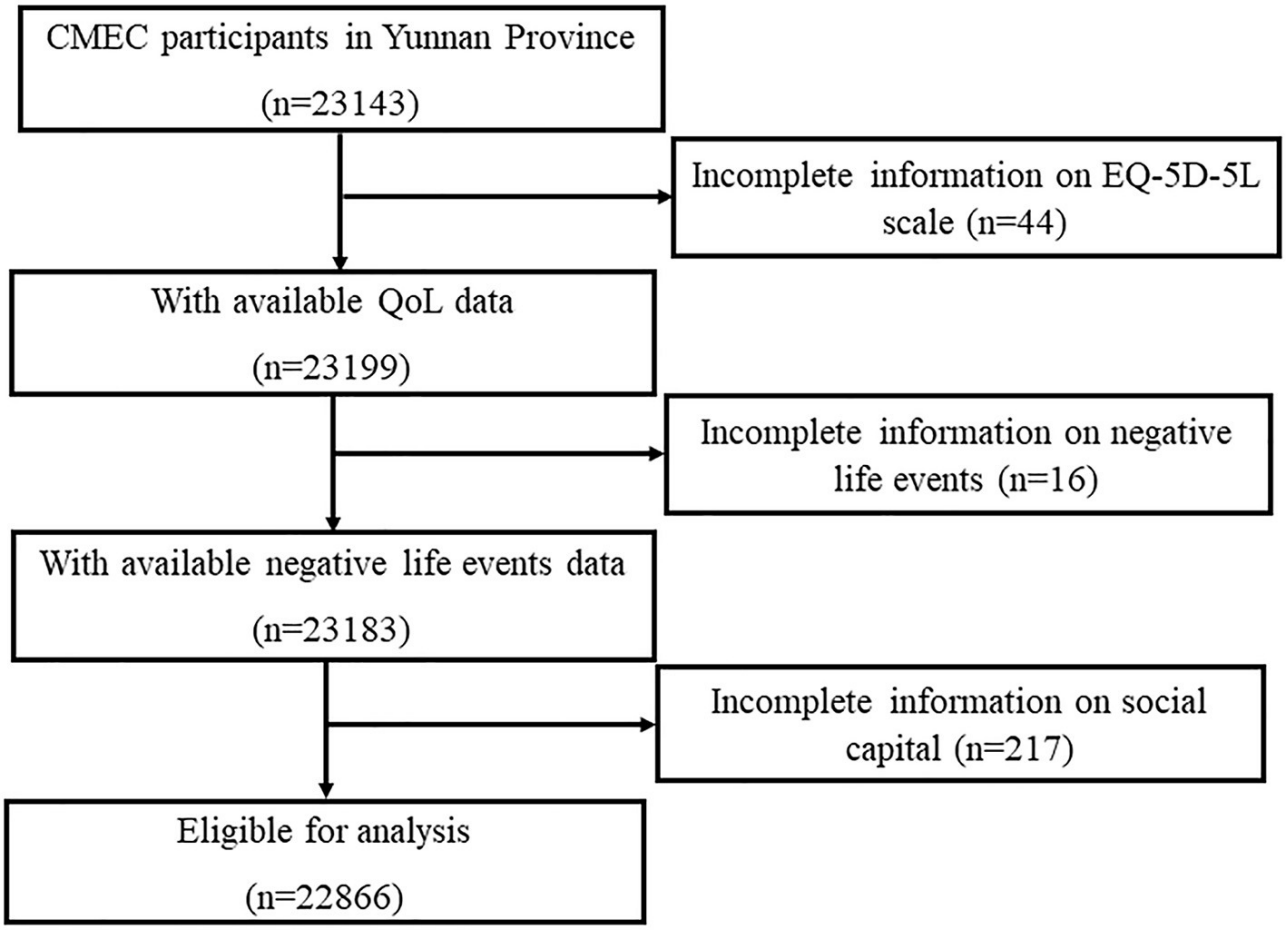
Flow diagram of selection of participant.

A total of 23,143 participants were enrolled in the Yunnan cohort, and 277 participants with incomplete information on social capital, NLE, QoL were excluded. Finally, 22,866 participants were ultimately included in this study (Figure 1). Power analysis revealed a power level of 1.00(*F* = 1.789, α = 0.05) in the present study (total sample size = 22,866, number of predictors = 11). This research received ethical approval from Kunming Medical University Medical Ethical Review Board (KMMU2020MEC078). On the day of data collection, the purpose and objectives of the study were explained to the respondents, and informed consent was obtained from each participant. The investigators used electronic questionnaires to conduct face-to-face surveys and they inputted the identity information of the respondents and recorded the whole interview process.

### Measured variables

#### Negative life events

Negative life events were measured by using a questionnaire developed by China Chronic Disease Prospective Research Project Group, that is, China Kadoorie Biobank Project Group which was the demonstration project for the CMEC (28). In this questionnaire, the participants were asked if they had experienced any of the following 10 life-changing events in the 2 years prior to the date of the survey: separation/divorce, unemployment/laid-off/retirement, business or family financial bankruptcy, violent assault/rape, serious family conflict, severe trauma or car accident, serious illness or death of spouse, death or illness of other family members, natural disasters and loss of financial resources/debt. In order to make the analysis more feasible and based on the thought that more NLE occurred, the greater impact on the community residents might be, we made the scoring rules for the NLE. Each question contained two choices: “Yes” and “No”. Each “Yes” response was worth 1 point, while “No” response was worth 0 points. The sum of the scores for the 10 questions is the NLE score. The score of NLE ranged from 0 to 10, and the higher the score, the more significant NLE the participant had experienced.

#### Social capital

In order to improve the reliability of responses and efficiency of investigation, we developed a five-items social capital scale that is simplified scale of the Chinese version of the Health-related Social Capital Scale (29). The five items of this brief scale were presented in the following order: (1) “Can your family offer you emotional (spiritual) support?”; (2) “When you need help, can your family offer you financial support?”; (3) “In the past year, have you frequently participated in activities, including parties, entertainments, and sports?”; (4) “When you are in a crisis or in need of help, can you always receive support from others?”; and (5) “Do you agree with the statement 'Most people who live near here can be trusted'?” The response values for each item ranged from 1 (never/strongly disagree) to 5 (always/strongly agree). The answer to each item was the score of the item, and the total score of social capital was the sum of the five items, so the total score of social capital ranged from 5 to 25. A higher score represents a higher level of social capital. When evaluating the reliability and validity of the social capital scale and analyzing the mediating effect of social capital, we used the item score. When assessing the association among the measured variables by using multiple linear regression analyses, we used the scale score.

#### Health-related quality of life

The EQ-5D-5L scale was used to measure the QoL of the participants. The scale is an instrument with a descriptive system and a visual analog scale (VAS). The descriptive system evaluated five dimensions of health status: mobility, self-care, daily activities, pain/discomfort and anxiety/depression. Each dimension had one item, and answer of each item was divided into five levels: no problems, slight problems, moderate problems, severe problems and extreme problems. The VAS records the respondents' self-rating for their current health state. This is a vertical thermometer-like scale, ranging from 0 (“worst imaginable health state”) to 100 (“best imaginable health state”), with higher scores for higher QoL. Respondents indicated where they perceived their current health status, relative to these anchor points. The Chinese version of the EQ-5D-5L scale was found to have good reliability and validity in the Chinese population (30). The Cronbach's α in this study was 0.760. There were two scoring methods for EQ-5D-5L scale. We could use a utility integral system conversion table (31), which converts respondents' choices in the five dimensions of QoL into EQ-5D index value, to assess the QoL of the participants. The EQ-5D index value ranges from 0 to 1, where higher scores stand for better QoL. We also could use the self-rating score (range from 0 to 100) from the respondents, named as EQ-VAS score. Both EQ-VAS score and EQ-5D index value can be used as indicators of quality of life. The difference between them is that EQ-VAS is the self-rating score of a single subject directly obtained from the survey, which is easy to obtain, sensitive and easy to understand. The EQ-5D index value is based on the overall population to evaluate the health status of individuals, which has the characteristics of good stability and strong comparability. In brief, they are different in terms of the measurement accuracy. In order to explore whether using the two indicators to represent the QoL would get different results, this study will use EQ-5D index value and EQ-VAS score as the QoL measurement indicators for the adults.

#### Other variables

In addition to the main research variables, we used a self-developed structured questionnaire to collect the socio-demographic information of the participants, including gender, age (30–39, 40–49, 50–59, 60–69, 70–79 years), ethnic group (Han, Yi, Bai), level of education (illiteracy, primary school, junior high school, high school and above), marital status (married/cohabitation, separated/divorced/unmarried, widowed), annual income(<12,000, 12,000–19,999, 20,000–59,999, 60,000–99,999, and ≥100,000 yuan), occupation (primary industry staff, secondary industry staff, tertiary industry staff, unemployed). The primary industry refers to the production of agricultural raw products, such as agriculture, forestry, etc. The secondary industry refers to the processing of raw materials provided by products, such as mining, manufacturing, etc. The tertiary industry refers to other industries except the primary industry and the secondary industry, mainly including transportation industry, catering industry, financial industry, etc.

### Theoretical framework

**Figure 2:**
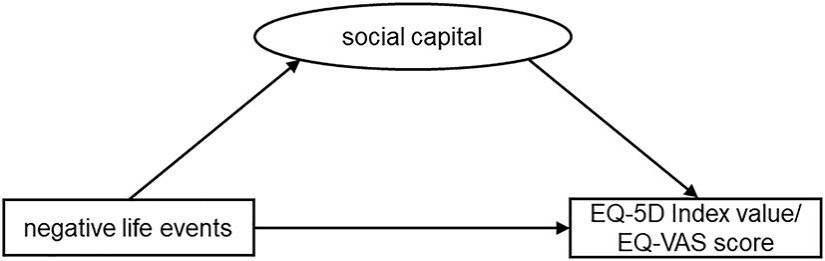
Theoretical model of the impact of social capital and negative life events on QoL of adults.

Stress buffering theory holds that social capital can buffer the negative effects of life events. Based on stress buffering theory and aforementioned literature analyses, we established the theoretical hypothesis that social capital plays a mediating role between NLE and QoL. It is hypothesized that negative life events would be associated with poorer social capital, and in turn poorer QoL (Figure 2). To be more specific, the following three hypotheses were proposed.

#### Hypothesis 1

There is a direct association between negative life events and QoL.

#### Hypothesis 2

There is an association between social capital and QoL.

#### Hypothesis 3

There is a specific indirect effect of negative life events on QoL through social capital.

### Statistical analyses

Firstly, descriptive statistics were conducted to show the demographic characteristics. Wilcoxon rank test or Kruskal-Wallis *H* test was used to test the differences in the distribution of NLE among different demographic characteristics. The distribution differences of social capital score or EQ-5D index value or EQ-VAS score in different demographic characteristics was examined by *t-*test or ANOVA.

Next, the reliability and validity of the self-developed social capital scale were evaluated by calculating the Cronbach's α coefficient and performing confirmatory factor analysis (CFA).

Then, to assess the association among the NLE, social capital and QoL, the correlation coefficients was calculated and the multiple linear regression analyses were conducted. To be specific, EQ-5D index value and EQ-VAS score were used as dependent variables respectively and the NLE score and social capital score were independent variables while some covariables including gender, age, education level and ethnicity, marital status, annual family income and occupation were adjusted in the regression model.

Finally, the mediating effect of social capital between NLE and QoL was explored by using structural equation model (SEM). Two mediation models were analyzed to examine social capital as mediator in relationship between NLE and EQ-5D index values, and in the relationship between NLE and EQ-VAS scores, respectively. To determine whether the standardize coefficient (β) was statistically significant, we used the bias-corrected bootstrap 95% confidence interval (95%CI) of β based on 5,000 bootstrap samples. The 95% CI of β did not contain zero, which indicated that the mediating effect or the direct effect was significant (*p* < 0.05).

In the confirmatory factor analysis and the structural equation model, the maximum likelihood (ML) method was used to estimate the model path coefficients and calculate the fitting statistics. Although ML assumes that the variables in the model are multivariate normally distributed, this normality assumption is difficult to meet in practice. Some scholars (32) believe that in most cases, even if the variables are not normally distributed, ML estimation is still reasonable for the estimation of each parameter. A set of goodness-of-fit indexes were calculated, including the chi-square (χ^2^), comparative fit index (CFI), incremental fix index (IFI), Tucker Lewis index (TLI) and root-mean square error of approximation (RMSEA). It can be considered that the model fits the sample data well and is acceptable when χ^2^ < 3.84, CFI ≥ 0.90, IFI ≥ 0.90, TLI ≥ 0.90, and RMSEA ≤ 0.08 (33).

All reported *p-*values were 2-tailed and α level of 0.05 or less was considered statistically significant. Statistical analyses were performed using SPSS version 20.0 and AMOS version 23.0.

To assess the robustness of our findings, we also performed several sensitivity analyses. First, the participants were, respectively, divided into different groups for subgroup analysis by sex (male, female), age (<60 years, ≥60 years), ethnic (Han, Bai, Yi), family income (<60,000 yuan, ≥60,000 yuan) and occupation (primary industry, others). According to the above grouping, we carried out mediating effect analyses for 11 times using SEM. Then, we classified negative life events into three categories: (1) family related events (couple separation/divorce/divorce, serious internal conflicts and rush, sudden, spouse death, death of other family members or serious illness); (2) economic related events (loss of financial source/debt, unemployment/layoffs/retirement, self-run business or family economic bankruptcy); (3) other events (hit/beaten by violence, severe trauma or car accident, serious natural disaster). We separately examined the mediating effect of social capital between the three categories of negative life events and QoL.

## Results

23,143 participants completed the survey, and 22,866 questionnaires were valid, achieving a response rate of 98.8%. Among the 22,866 participants included in this study, 33.1% (*n* = 7,568) were male, and 66.9% were female (*n* = 15,298). The mean age of the subjects was 52.60 ± 10.48 years old, and the age of most subjects (62.7%, *n* = 14,337) ranged from 40 to 59 years old. There were participants of three different nationalities: 45.9% (*n* = 10,485) were of Han nationality, 27.4% (*n* = 6,274) were of Yi nationality, and 26.7% (*n* = 6,107) were of Bai nationality. Approximately 29.0% (*n* = 6,635) had attended school informally, 39.0% (*n* = 8,925) had attended primary school, 24.4% (*n* = 5,582) had attended junior high school, and only 7.5% (*n* = 1,722) had attended high school and above. Overall, 89.7% of participants (*n* = 20,513) were married status, 67.3% of participants (*n* = 15,391) were engaged in the primary industry, and the vast majority of the participants had the annual household income of 12,000~59,999 yuan. The mean of EQ-5D index value was 0.92 (SD = 0.12), and the mean of EQ-VAS score was 71.77 (SD = 13.80), and the mean of social capital score was 19.82 (SD = 3.24). The EQ-5D index values and EQ-VAS scores were statistically significant in the distribution of all socio-demographic characteristics. Univariate analysis showed that gender, age, ethnic, marital status, education level, annual household income and occupation were significantly related to the QoL (*p* < 0.05). The demographic data on study participants and the comparison of the key measurement variables in different sample characteristics were presented in Table 1.

**Table 1:** The comparison of the key measurement variables in different sample characteristics.

**Characteristic**	***n* (*%*)**	**SC score**	**NLE score**	**EQ-5D index value**	**EQ-VAS score**
**Gender**
Male	7,568 (33.1)	19.87 ± 3.23	0.0 (0.0, 1.0)	0.94 ± 0.11	72.98 ± 13.84
Female	15,298 (66.9)	19.80 ± 3.24	0.0 (0.0, 1.0)	0.92 ± 0.12	71.17 ± 13.74
*p*-value	-	0.115	0.002	<0.001	*p* < 0.001
**Age**
30~	2,378 (10.4)	20.03 ± 2.93	0.0 (0.0, 1.0)	0.97 ± 0.06	77.34 ± 12.40
40~	6,800 (29.7)	19.75 ± 3.12	0.0 (0.0, 1.0)	0.95 ± 0.09	73.91 ± 13.34
50~	7,537 (33.0)	19.80 ± 3.20	0.0 (0.0, 1.0)	0.92 ± 0.12	70.87 ± 13.78
60~	4,666 (20.4)	19.88 ± 3.42	0.0 (0.0, 1.0)	0.89 ± 0.14	69.00 ± 13.75
70~79	1,482 (6.5)	19.73 ± 3.48	0.0 (0.0, 1.0)	0.86 ± 0.17	66.26 ± 13.58
*p*-value	-	0.003	0.148	< 0.001	< 0.001
**Ethnic**
Han	10,485 (45.9)	19.50 ± 3.31	0.0 (0.0, 1.0)	0.94 ± 0.10	72.29 ± 14.13
Yi	6,274 (27.4)	19.28 ± 3.31	0.0 (0.0, 1.0)	0.88 ± 0.15	67.66 ± 13.43
Bai	6,107 (26.7)	20.93 ± 2.73	0.0 (0.0, 0.0)	0.94 ± 0.10	75.09 ± 12.50
*p*-value	-	<0.001	<0.001	<0.001	<0.001
**Educational level**
Non-formal schooling	6,635 (29.0)	19.56 ± 3.42	0.0 (0.0, 1.0)	0.89 ± 0.14	69.05 ± 13.90
Primary	8,925 (39.0)	19.77 ± 3.24	0.0 (0.0, 1.0)	0.92 ± 0.12	71.52 ± 13.73
Junior high school	5,582 (24.4)	20.04 ± 3.04	0.0 (0.0, 1.0)	095 ± 0.09	74.09 ± 13.34
High school and above	1,723 (7.5)	20.38 ± 2.96	0.0 (0.0, 1.0)	0.96 ± 0.08	76.09 ± 12.81
*p*-value	-	<0.001	0.158	<0.001	<0.001
**Marital status**
Married/cohabitation	20,513 (89.7)	19.89 ± 3.19	0.0 (0.0, 1.0)	0.93 ± 0.11	72.21 ± 13.67
Separated/divorced/unmarried	561 (2.5)	18.65 ± 3.63	0.0 (0.0, 1.0)	0.91 ± 0.14	70.44 ± 15.19
Widowed	1,791 (7.8)	19.41 ± 3.55	0.0 (0.0, 1.0)	0.86 ± 0.16	67.18 ± 13.89
*p*-value	-	<0.001	<0.001	<0.001	<0.001
**Annual household income (**¥**)**
<12,000	4,779 (20.9)	18.99 ± 3.68	0.0 (0.0, 1.0)	0.88 ± 0.15	67.86 ± 14.46
12,000~59,999	14,817 (64.9)	19.87 ± 3.11	0.0 (0.0, 1.0)	0.93 ± 0.11	72.05 ± 13.45
60,000~99,999	1,853 (8.1)	20.69 ± 2.85	0.0 (0.0, 0.0)	0.95 ± 0.07	75.66 ± 12.89
≥100,000	1,386 (6.1)	21.03 ± 2.63	0.0 (0.0, 0.0)	0.96 ± 0.07	77.20 ± 12.47
*p*-value	-	<0.001	<0.001	<0.001	<0.001
**Occupation**
Primary industry	15,391 (67.3)	19.75 ± 3.19	0.0 (0.0, 1.0)	0.92 ± 0.12	71.08 ± 13.73
Secondary industry	762 (3.3)	19.53 ± 3.31	0.0 (0.0, 1.0)	0.96 ± 0.07	75.53 ± 12.80
Tertiary industry	5,881 (25.7)	20.02 ± 3.32	0.0 (0.0, 1.0)	0.93 ± 0.12	73.10 ± 1,396
Unemployed	828 (3.6)	19.91 ± 3.32	0.0 (0.0, 1.0)	0.92 ± 0.11	71.62 ± 13.66
*p*-value	-	<0.001	<0.001	<0.001	<0.001

NLE, negative life events; SC, social capital.

For NLE score, the median is outside the parentheses and the interquartile are in parentheses.

The primary industry refers to the production of agricultural raw products, such as agriculture, forestry, etc. The secondary industry refers to the processing of raw materials provided by products, such as mining, manufacturing, etc. The tertiary industry refers to other industries except the primary industry and the secondary industry, mainly including transportation industry, catering industry, financial industry, etc.

The Cronbach's α coefficient value of the social capital scale in this study was 0.612. According to the Cronbach's α coefficient, the reliability of a scale can be divided into the following five categories: very low (α ≤ 0.30); low (0.30 < α ≤ 0.60); moderate (0.60 < α ≤ 0.75); high (0.75 < α ≤ 0.90) and very high (α > 0.90) (34). It can be considered that the reliability of the brief social capital scale was moderate. In the initial model (Figure S1 in Appendix), it was assumed that the error terms were independent of each other, and there was no correlation among the error terms of each measurement index. Confirmatory factor analysis showed that χ^2^ = 1479.60, *p* < 0.001, RMSEA = 0.114, CFI = 0.907, IFI = 0.907, and TLI = 0.814. The model goodness-of-fit statistics showed that the theoretical model was unacceptable. The model modification index and the expected parameter change value showed that the error terms were not independent of each other and that there may have been some relationship among the error terms. Therefore, the correlations among the error terms were added one by one to the model. The results of the modified model (Figure S2 in Appendix) showed that χ^2^ = 51.82, *p* < 0.001; RMSEA = 0.027, which was < 0.08; and CFI = 0.997, IFI = 0.997, and TLI = 0.990, all of which were > 0.9. Thus, the modified model was considered acceptable. The factor loading of the measured variables on the potential variables were significant (*p* < 0.05), which indicated that the measured variables responded well to latent variables. The structural validity of the brief social capital scale is good.

The correlation analysis among NLE, social capital, EQ-5D index value and EQ-VAS score showed that NLE were negatively correlated with social capital (*r* = −0.123, *p* < 0.001), or EQ-5D index value (*r* = −0.116, *p* < 0.001), or EQ-VAS score (*r* = −0.139, *p* < 0.001), respectively. There was positive association between social capital and EQ-5D index value (*r* = 0.129, *p* < 0.001), as well as between social capital and EQ-VAS score (*r* = 0.145, *p* < 0.001). As shown in Table 2, t after some covariates (gender, age, education level and ethnicity, marital status, annual family income and occupation) were controlled, both NLE was negatively associated with EQ-5D index value and EQ-VAS score in the multiple linear regression analyses. It was indicated that individuals with more NLE had a worse QoL.

**Table 2:** Regression analyses on the association among main study variables.

	**EQ-5D index value**		**EQ-VAS score**	
	**Crude**	**Adjusted**	**Crude**	**Adjusted**
	**model**	**model^#^**	**model**	**model^#^**
NLE	−0.023***	−0.018***	−2.866***	−2.468***
SC	0.004***	0.003***	0.538***	0.457***

The numbers in this table are regression coefficients (* means the *p*-value is smaller than 0.05; ** means the *p*-value is smaller than 0.01;

*** means the *p*-value is smaller than 0.001).

NLE, negative life events; SC, social capital.

# Adjusted model: adjusted for demographic features (gender, age education level and ethnicity, marital status, annual household income and occupation).

**Figure 3:**
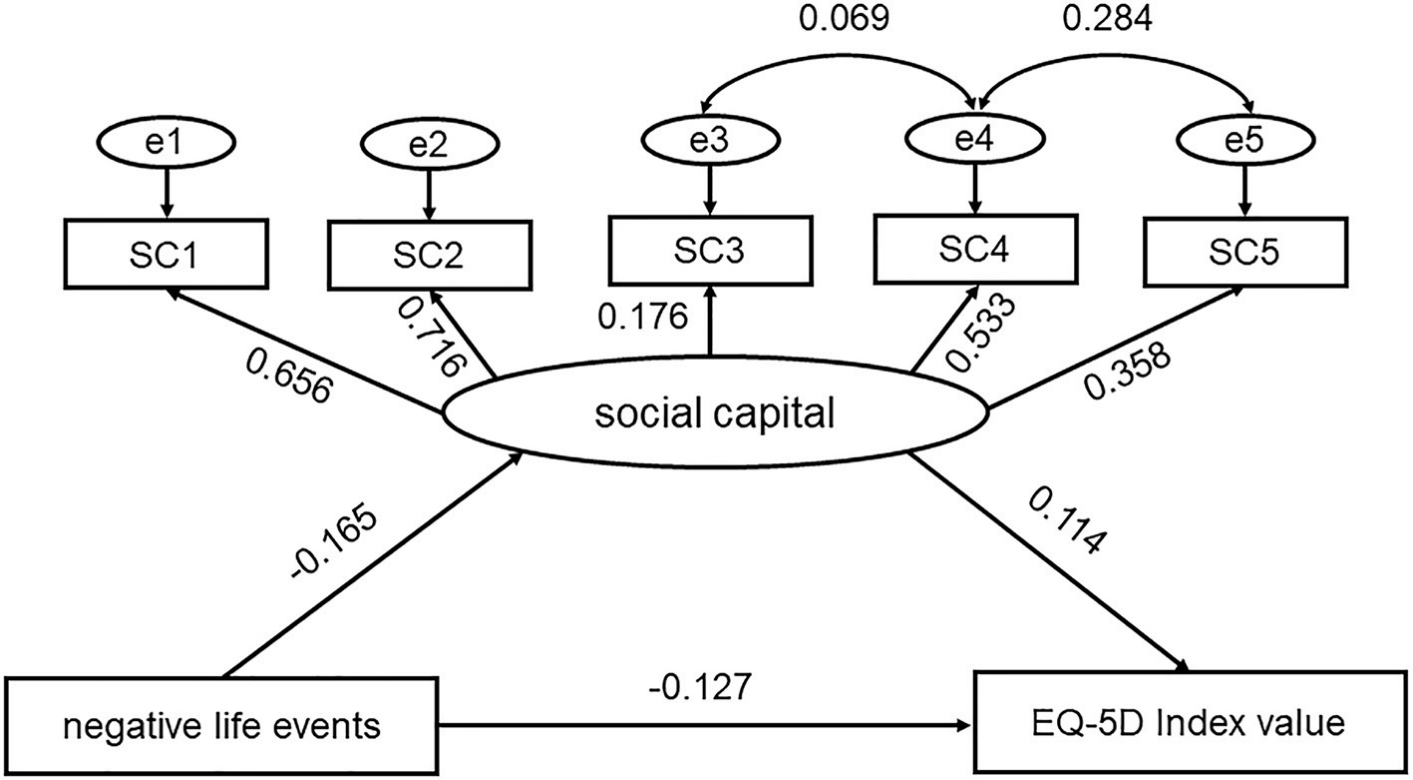
Conceptual model of the impact of social capital and negative life events on EQ-5D index value. SC1~SC5 represent five items in the brief social capital scale.

**Figure 4:**
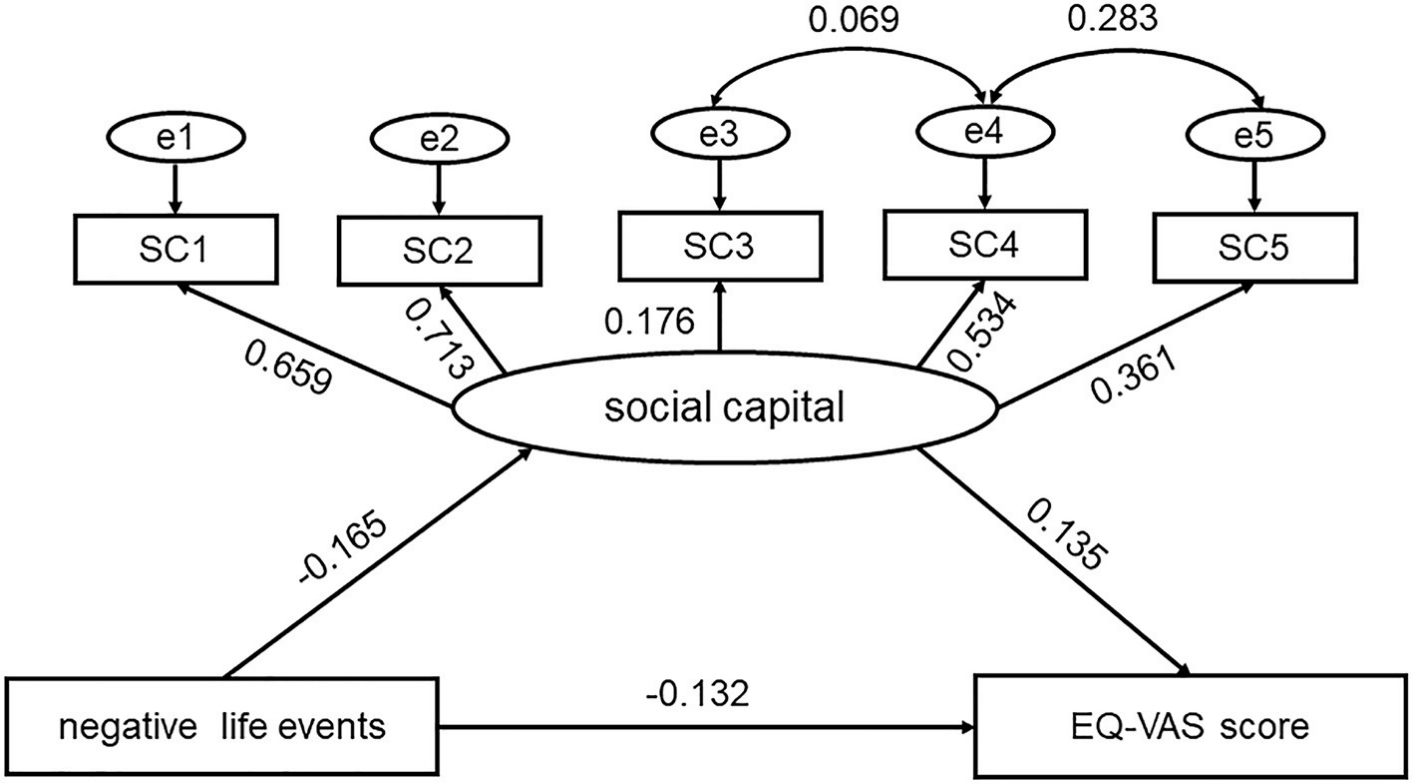
Conceptual model of the impact of social capital and negative life events on EQ-VAS score. SC1~SC5 represent five items in the brief social capital scale.

A separate model was created for each indicator of QoL (EQ-5D index value and EQ-VAS score). When we used EQ-5D index value to reflect QoL, the results of the SEM showed that χ^2^ = 268.53, *p* < 0.001, RMSEA = 0.032, CFI = 0.985, IFI = 0.985, and TLI = 0.971. When we used EQ-VAS score to reflect QoL, the results of the SEM showed that χ^2^ = 267.83, *p* < 0.001, RMSEA = 0.032, CFI = 0.985, IFI = 0.985, and TLI = 0.972. The two models and their corresponding standardized path coefficients are shown in Figures 3, 4 and Table 3. The goodness-of-fit statistics of the two models showed that the constructed theoretical models fit the data well and were consistent with the theoretical hypothesis.

**Table 3:** Direct effects, indirect effects, and the corresponding 95% confidence intervals between the study variables.

		**95% CI**	
**Model pathways**	**Standardized**	**Lower**	**Upper**
	**coefficient**	**bounds**	**bounds**
**The effect of NLE on EQ-5D index value**			
**Direct effect**			
NLE → SC	−0.165	−0.183	−0.147
SC → EQ-5D index value	0.114	0.097	0.133
NLE → EQ-5D index value	−0.127	−0.144	−0.110
**Indirect effect**			
NLE → SC → EQ-5D index value	−0.019	−0.023	−0.015
**The effect of NLE on EQ-VAS score**			
**Direct effect**			
NLE → SC	−0.165	−0.183	−0.147
SC → EQ-VAS score	0.135	0.117	0.151
NLE → EQ-VAS score	−0.132	−0.146	−0.118
**Indirect effect**			
NLE → SC → EQ-VAS score	−0.022	−0.026	−0.019

NLE, negative life events; SC, social capital.

As shown in Table 3, NLE not only directly negatively affected EQ-5D index value [β = −0.127, 95% CI (−0.144, −0.110)] but also indirectly negatively affected EQ-5D Index value through social capital [β = −0.019, 95% CI (−0.023, −0.015)]. Social capital had a direct positive effect on EQ-5D index value [β = 0.114, 95% CI (0.097, 0.133)]. Therefore, the mediating effect of the social capital was significant and social capital could buffer the negative impact of life events on EQ-5D Index value. The social capital-mediated indirect effect accounted for 10.3% of the total effect when using EQ-5D index value to represent QoL.

Similarly, the direct effect of NLE on EQ-VAS score was negatively significant [β = −0.132, 95% CI (−0.146, −0.118)]. The indirect effect of NLE on EQ-VAS score through social capital also negatively significant [β = −0.022, 95% CI (−0.026, −0.019)]. However, the social capital positively affected EQ-VAS score [β = 0.135, 95% CI (0.117, 0.151)]. Therefore, we could think social capital could buffer the negative impact of life events on EQ-VAS score. The social capital-mediated indirect effect accounted for 11.8% of the total effect when using EQ-VAS score represent QoL.

In the sensitivity analyses, similar findings were found. The social capital still played a mediating effect between NLE and QoL and still could buffer the negative impact of life events on QoL in different subgroups (Supplementary Tables S1–S11). For different types of life events, the mediating effect of social capital was still significant (Supplementary Tables S12–S14). The above results were presented in the Data Sheet.

## Discussion

The aim of present study was to examine the mediating effect of social capital between NLE and QoL in a sample of community adults from Yunnan province, China. Therefore, we established the theoretical hypothesis that social capital plays a mediating role between NLE and QoL. It is hypothesized that NLE would be associated with poorer social capital, and in turn poorer QoL. Based on the hypothesis, mediation analysis was conducted with social capital as a mediating variable by SEM. The results of SEM showed that negative life events were negatively correlated with social capital and QoL and that social capital played a mediating role in the relationship between life events and QoL.

No matter using EQ-5D index value or EQ-VAS score to represent QoL, we found that NLE not only had a direct negative impact on QoL but also had an indirect impact on QoL through the mediating effect of social capital in adults. On the one hand, the social capital was negatively correlated with NLE. NLE may influence social capital by changing the structural and functional characteristics of an individual's social capital system. For example, elderly people who had experienced bereavement were found to have fewer sources of social support, and the scale of their social support networks decreased, thus affecting the availability of social resources. One study found that natural disasters could weaken the ability of social capital systems at both the individual level and the community level (35). Actually, it was a vicious circle between NLE and social capital. NLE could weaken the individual social capital system; in turn, insufficient social capital would increase the risk of NLE (36–38). On the other hand, the social capital (including emotional support, financial support, etc.) received from family members or others played a positive role in the QoL of the participating Chinese adults. This was consistent with previous research results (39–44). In the context of Chinese culture, family is the main component of the social environment, and family support plays an important role in the lives of Chinese people. Good family financial and emotional support can promote individual mental health and improve QoL. As a kind of social resource, social capital is beneficial to provide access to social support, promote health protective behaviors, reduce the risk of loneliness and depression, increase satisfaction and happiness, and improve QoL. Individuals with higher social capital are more inclined to actively participate in social activities, strengthen social interaction between individuals and members of other groups in the process of activities, and form a more stable social network. In this social network, individuals have access to appropriate resources such as help, support and services, providing effective access for older adults to social resources and information about health behaviors (45, 46). In addition, good social capital can also promote cooperation and help among community members, increase security and subjective well-being, and improve individual mental health.

In other words, the social capital had a significant mediating effect between life events and QoL, suggesting that social capital could buffer the negative impact of life events on QoL. A similar study (47) pointed out that the social support could improve the emotional experience of individuals for the life events and played a psychological buffer effect on NLE although the quality of life of the elderly was affected by NLE to a certain extent. As a source of stress, NLE stimulate the brain's stress assessment and cause a series of stress responses in the body, which affect the individual's health. When a life event is assessed by the brain as a threat to an individual, it stimulates the body's emotional response (such as anxiety and depression), causes a series of physiological responses or behavioral changes, promotes the occurrence and development of diseases, and thus affects the QoL of an individual. However, social capital, as a kind of external social resource, could improve individuals' emotional experience with life events and encourage individuals to take positive coping styles to face the negative influence of life events. For example, a study on depression in Chinese elderly (48) found that family social capital could alleviate the negative effect of NLE on depression in the elderly. The findings in this study had important applications for improving the QoL of residents. Social capital was an effective resource for adults to cope with NLE. For adults who suffer from major life events, the role of social capital should be emphasized, and the establishment of a good interpersonal network should be encouraged.

The work had some limitations. First, it is necessary to be cautious when extending the conclusions to other people with specific cultural contexts because the study object was from the adults living in Yunnan Province, China. Second, this was a cross-sectional survey, and the causal model was deduced based on theory, so causal inferences could not be made. In future studies, longitudinal data could be obtained with the help of cohort follow-up to provide more definitive information for causal inference. Third, there is overriding factor that was not evaluated in this study, which may also mediate the association between NLE and QoL, which is the sense of coherence (SOC). SOC is defined as a global orientation that allows people to manage stress, identify their internal and external environments and find solutions for their health (49). The high SOC could reduce the level of experienced stress, weaken the negative effects of NLE, and maintain the health of individuals encountering life events (49, 50). In future study, we should evaluate this factor to deeply explore the mechanism of influencing NLE on the QoL. Finally, the brief social capital scale is so simple, fast and convenient that it is easily accepted by respondents. However, the internal consistency of the scale is not good enough. In future research work, the brief social capital scale needs to be further revised and improved.

## Conclusion

In summary, NLE could reduce the QoL of the adults in China and social capital played a mediating role in the relationship between NLE and QoL, which could alleviate the negative impact of NLE on QoL of the adults in China. Families and society should pay special attention to people who experience NLE, provide better social capital for them, and improve their QoL.

## Data availability statement

The original contributions presented in the study are included in the article/Supplementary material, further inquiries can be directed to the corresponding authors.

## Ethics statement

The ethical approval from Kunming Medical University Medical Ethical Review Board (KMMU2020MEC078). The patients/participants provided their written informed consent to participate in this study.

## Author contributions

JZ and SW analyzed and interpreted the data and drafted the manuscript, which was critically revised by all others. XZ, XH, HD, NC, YS, CS, and ZH contributed to the data collection and data analysis. QM and JY contributed to the design of the study and the supervision of the statistical analysis. QM revised the manuscript and had primary responsibility for the final content. All authors have read and agreed to the published version of the manuscript.

## Funding

This study was funded by the National Natural Science Foundation of China (No. 81960617) and Yunnan Applied Basic Research Projects-Kunming Medical University Union Foundation (No. 202001AY070001-182). The study funder had no influence on the study design, collection, management, analysis, and interpretation of data, writing of the report, or decision to submit the report for publication.

## Conflict of interest

The authors declare that the research was conducted in the absence of any commercial or financial relationships that could be construed as a potential conflict of interest.

## Publisher's note

All claims expressed in this article are solely those of the authors and do not necessarily represent those of their affiliated organizations, or those of the publisher, the editors and the reviewers. Any product that may be evaluated in this article, or claim that may be made by its manufacturer, is not guaranteed or endorsed by the publisher.
