# Supplementary material for: Mediating role of social capital on the association between negative life events and quality of life among adults in China: A population-based study
Source: Front Public Health. 2022 Sep 29;10:987579. doi: 10.3389/fpubh.2022.987579 (PMC9557146; doi:10.3389/fpubh.2022.987579)
Supplement: Supplementary file 1 [file Data_Sheet_1.pdf]

Appendix

About the results of CFA for the social capital scale

Confirmatory factor analysis was used to assess the validity of social capital scale. In the initial model (**Figure S1**), it was assumed that the error terms were independent of each other, and there was no correlation among the error terms of each measurement index. Confirmatory factor analysis showed that χ^2 =1479.60, p<0.001, RMSEA=0.114, CFI=0.907, IFI=0.907, and TLI=0.814. The model goodness-of-fit statistics showed that the theoretical model was unacceptable.

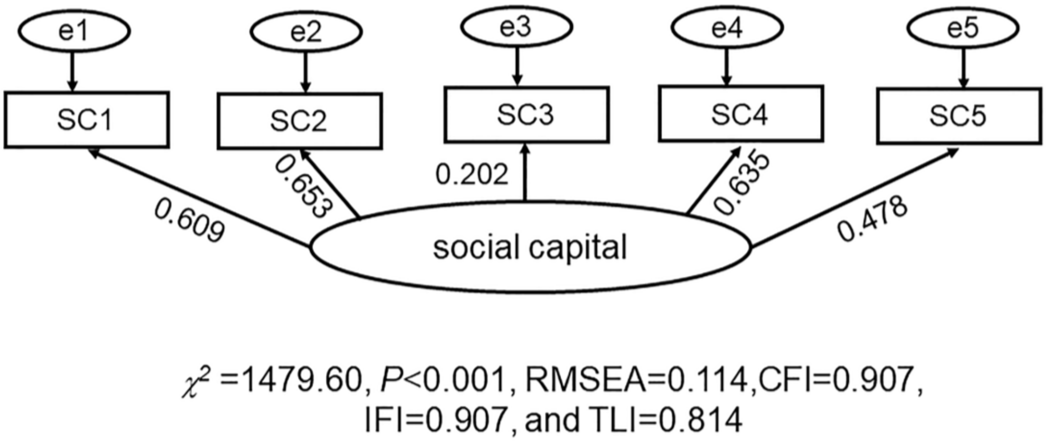

Figure S1 Initial conceptual model of the social capital.

Note: SC1~SC5 represent five items in the ultra-brief social capital scale.

The results of the modified model (**Figure S2**) showed that χ^2 =51.82, p<0.001; RMSEA=0.027, which was less than 0.08; and CFI=0.997, IFI=0.997, and TLI=0.990, all of which were > 0.9. Thus, the modified model was considered acceptable.

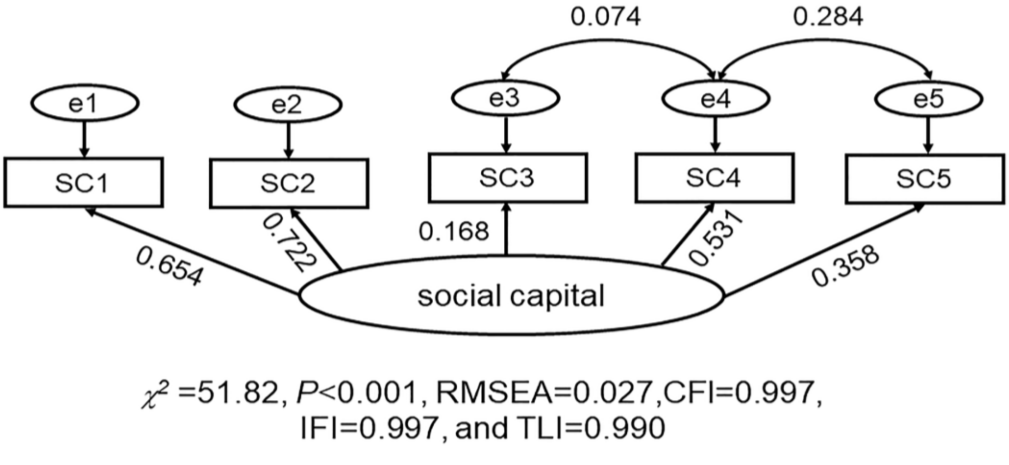

Figure S2 Modified model of the social capital.

*Note:*SC1~SC5 represent five items in the ultra-brief social capital scale

The results of sensitivity analyses

To assess the robustness of our findings, we also performed the following sensitivity analyses.

On the one hand, the participants were respectively divided into different groups for subgroup analysis by sex (male, female), age (<60 years, ≥60 years), ethnic (Han, Bai, Yi), family income (<60000 yuan, ≥60000 yuan) and occupation (primary industry, others). The primary industry refers to the production of agricultural raw products, such as agriculture, forestry, etc. According to the above grouping, we carried out mediating effect analyses for 11 times using SEM.It was found that social capital still played a mediating effect between negative life events and QoL across subgroups of gender, age, ethnicity, household income, and occupation (**Table S1~Table S11**).

Table S1 Direct effects, indirect effects, and the corresponding 95% confidence intervals between the study variables in the male group

Model pathways	Standardized coefficient	95% CI	
		Lower bounds	Upper bounds
The effect of NLE on EQ-5D index value			
**Direct effect**			
NLE→SC	-0.134	-0.167	-0.102
SC→EQ-5D index value	0.097	0.067	0.130
NLE→EQ-5D index value	-0.116	-0.147	-0.086
**Indirect effect**			
NLE→SC→EQ-5D index value	-0.013	-0.020	-0.008
The effect of NLE on EQ-VAS score			
**Direct effect**			
NLE→SC	-0.134	-0.167	-0.102
SC→EQ-VAS score	0.116	0.086	0.145
NLE→EQ-VAS score	-0.126	-0.152	-0.101
**Indirect effect**			
NLE→SC→EQ-VAS score	-0.015	-0.022	-0.010

Note: NLE, negative life events; SC, social capital

Table S2 Direct effects, indirect effects, and the corresponding 95% confidence intervals between the study variables in the female group

Model pathways	Standardized coefficient	95% CI	
		Lower bounds	Upper bounds
The effect of NLE on EQ-5D index value			
**Direct effect**			
NLE→SC	-0.178	-0.200	-0.155
SC→EQ-5D index value	0.123	0.101	0.145
NLE→EQ-5D index value	-0.129	-0.150	-0.110
**Indirect effect**			
NLE→SC→EQ-5D index value	-0.022	-0.027	-0.017
The effect of NLE on EQ-VAS score			
**Direct effect**			
NLE→SC	-0.178	-0.200	-0.155
SC→EQ-VAS score	0.144	0.123	0.165
NLE→EQ-VAS score	-0.133	-0.150	-0.117
**Indirect effect**			
NLE→SC→EQ-VAS score	-0.026	-0.031	-0.021

Note: NLE, negative life events; SC, social capital

Table S3 Direct effects, indirect effects, and the corresponding 95% confidence intervals between the study variables in the younger adult group

Model pathways	Standardized coefficient	95% CI	
		Lower bounds	Upper bounds
The effect of NLE on EQ-5D index value			
**Direct effect**			
NLE→SC	-0.151	-0.171	-0.129
SC→EQ-5D index value	0.108	0.087	0.130
NLE→EQ-5D index value	-0.127	-0.147	-0.107
**Indirect effect**			
NLE→SC→EQ-5D index value	-0.016	-0.021	-0.012
The effect of NLE on EQ-VAS score			
**Direct effect**			
NLE→SC	-0.151	-0.171	-0.129
SC→EQ-VAS score	0.132	0.112	0.152
NLE→EQ-VAS score	-0.134	-0.150	-0.117
**Indirect effect**			
NLE→SC→EQ-VAS score	-0.020	-0.024	-0.016

Note: NLE, negative life events; SC, social capital

Table S4 Direct effects, indirect effects, and the corresponding 95% confidence intervals between the study variables in the elderly group

Model pathways	Standardized coefficient	95% CI	
		Lower bounds	Upper bounds
The effect of NLE on EQ-5D index value			
**Direct effect**			
NLE→SC	-0.196	-0.231	-0.162
SC→EQ-5D index value	0.130	0.096	0.164
NLE→EQ-5D index value	-0.128	-0.159	-0.097
**Indirect effect**			
NLE→SC→EQ-5D index value	-0.025	-0.035	-0.018
The effect of NLE on EQ-VAS score			
**Direct effect**			
NLE→SC	-0.196	-0.231	-0.162
SC→EQ-VAS score	0.143	0.110	0.174
NLE→EQ-VAS score	-0.124	-0.152	-0.097
**Indirect effect**			
NLE→SC→EQ-VAS score	-0.028	-0.037	-0.020

Note: NLE, negative life events; SC, social capital

Table S5 Direct effects, indirect effects, and the corresponding 95% confidence intervals between the study variables in the Han group

Model pathways	Standardized coefficient	95% CI	
		Lower bounds	Upper bounds
The effect of NLE on EQ-5D index value			
**Direct effect**			
NLE→SC	-0.165	-0.191	-0.138
SC→EQ-5D index value	0.077	0.052	0.105
NLE→EQ-5D index value	-0.110	-0.134	-0.088
**Indirect effect**			
NLE→SC→EQ-5D index value	-0.013	-0.018	-0.008
The effect of NLE on EQ-VAS score			
**Direct effect**			
NLE→SC	-0.165	-0.191	-0.138
SC→EQ-VAS score	0.091	0.067	0.116
NLE→EQ-VAS score	-0.118	-0.139	-0.098
**Indirect effect**			
NLE→SC→EQ-VAS score	-0.015	-0.021	-0.011

Note: NLE, negative life events; SC, social capital

Table S6 Direct effects, indirect effects, and the corresponding 95% confidence intervals between the study variables in the Bai group

Model pathways	Standardized coefficient	95% CI	
		Lower bounds	Upper bounds
The effect of NLE on EQ-5D index value			
**Direct effect**			
NLE→SC	-0.147	-0.190	-0.106
SC→EQ-5D index value	0.155	0.117	0.198
NLE→EQ-5D index value	-0.082	-0.117	-0.048
**Indirect effect**			
NLE→SC→EQ-5D index value	-0.023	-0.034	-0.015
The effect of NLE on EQ-VAS score			
**Direct effect**			
NLE→SC	-0.147	-0.190	-0.106
SC→EQ-VAS score	0.143	0.104	0.181
NLE→EQ-VAS score	-0.102	-0.131	-0.073
**Indirect effect**			
NLE→SC→EQ-VAS score	-0.021	-0.030	-0.014

Note: NLE, negative life events; SC, social capital

Table S7 Direct effects, indirect effects, and the corresponding 95% confidence intervals between the study variables in the Yi group

Model pathways	Standardized coefficient	95% CI	
		Lower bounds	Upper bounds
The effect of NLE on EQ-5D index value			
**Direct effect**			
NLE→SC	-0.089	-0.121	-0.057
SC→EQ-5D index value	0.105	0.072	0.139
NLE→EQ-5D index value	-0.122	-0.153	-0.092
**Indirect effect**			
NLE→SC→EQ-5D index value	-0.009	-0.015	-0.005
The effect of NLE on EQ-VAS score			
**Direct effect**			
NLE→SC	-0.089	-0.121	-0.057
SC→EQ-VAS score	0.122	0.092	0.154
NLE→EQ-VAS score	-0.115	-0.141	-0.089
**Indirect effect**			
NLE→SC→EQ-VAS score	-0.011	-0.016	-0.007

Note: NLE, negative life events; SC, social capital

Table S8 Direct effects, indirect effects, and the corresponding 95% confidence intervals between the study variables in the primary industry group

Model pathways	Standardized coefficient	95% CI	
		Lower bounds	Upper bounds
The effect of NLE on EQ-5D index value			
**Direct effect**			
NLE→SC	-0.150	-0.172	-0.128
SC→EQ-5D index value	0.114	0.092	0.136
NLE→EQ-5D index value	-0.115	-0.136	-0.096
**Indirect effect**			
NLE→SC→EQ-5D index value	-0.017	-0.022	-0.013
The effect of NLE on EQ-VAS score			
**Direct effect**			
NLE→SC	-0.150	-0.172	-0.128
SC→EQ-VAS score	0.126	0.105	0.146
NLE→EQ-VAS score	-0.126	-0.143	-0.109
**Indirect effect**			
NLE→SC→EQ-VAS score	-0.019	-0.024	-0.015

Note: NLE, negative life events; SC, social capital

Table S9 Direct effects, indirect effects, and the corresponding 95% confidence intervals between the study variables in other occupation group

Model pathways	Standardized coefficient	95% CI	
		Lower bounds	Upper bounds
The effect of NLE on EQ-5D index value			
**Direct effect**			
NLE→SC	-0.192	-0.222	-0.161
SC→EQ-5D index value	0.113	0.081	0.147
NLE→EQ-5D index value	-0.153	-0.184	-0.123
**Indirect effect**			
NLE→SC→EQ-5D index value	-0.022	-0.030	-0.015
The effect of NLE on EQ-VAS score			
**Direct effect**			
NLE→SC	-0.191	-0.222	-0.161
SC→EQ-VAS score	0.152	0.124	0.181
NLE→EQ-VAS score	-0.1148	-0.172	-0.122
**Indirect effect**			
NLE→SC→EQ-VAS score	-0.029	-0.037	-0.022

Note: NLE, negative life events; SC, social capital

Table S10 Direct effects, indirect effects, and the corresponding 95% confidence intervals between the study variables in the “low family income level” group

Model pathways	Standardized coefficient	95% CI	
		Lower bounds	Upper bounds
The effect of NLE on EQ-5D index value			
**Direct effect**			
NLE→SC	-0.158	-0.178	-0.138
SC→EQ-5D index value	0.105	0.085	0.124
NLE→EQ-5D index value	-0.128	-0.146	-0.111
**Indirect effect**			
NLE→SC→EQ-5D index value	-0.017	-0.021	-0.013
The effect of NLE on EQ-VAS score			
**Direct effect**			
NLE→SC	-0.158	-0.178	-0.138
SC→EQ-VAS score	0.125	0.106	0.143
NLE→EQ-VAS score	-0.135	-0.150	-0.120
**Indirect effect**			
NLE→SC→EQ-VAS score	-0.020	-0.024	-0.016

Note: NLE, negative life events; SC, social capital

Table S11 Direct effects, indirect effects, and the corresponding 95% confidence intervals between the study variables in the "high family income level" group

Model pathways	Standardized coefficient	95% CI	
		Lower bounds	Upper bounds
The effect of NLE on EQ-5D index value			
**Direct effect**			
NLE→SC	-0.154	-0.211	-0.101
SC→EQ-5D index value	0.080	0.028	0.134
NLE→EQ-5D index value	-0.065	-0.114	-0.020
**Indirect effect**			
NLE→SC→EQ-5D index value	-0.012	-0.024	-0.004
The effect of NLE on EQ-VAS score			
**Direct effect**			
NLE→SC	-0.154	-0.211	-0.101
SC→EQ-VAS score	0.098	0.051	0.145
NLE→EQ-VAS score	-0.064	-0.107	-0.024
**Indirect effect**			
NLE→SC→EQ-VAS score	-0.015	-0.026	-0.007

Note: NLE, negative life events; SC, social capital

On the other hand, we classified negative life events into three categories: (1) family related events (couple separation / divorce / divorce, serious internal conflicts and rush, sudden, spouse death, death of other family members or serious illness); (2) economic related events (loss of financial source / debt, unemployment / layoffs / retirement, self-run business or family economic bankruptcy); (3) other events (hit / beaten by violence, severe trauma or car accident, serious natural disaster). We separately examined the mediating effect of social capital between the three categories of negative life events and QoL. For different types of life events, we found that the mediating effect of social capital was still significant (**Table S12~Table S14**).

Table S12 Direct effects, indirect effects, and the corresponding 95% confidence intervals between the study variables

Model pathways	Standardized coefficient	95% CI	
		Lower bounds	Upper bounds
The effect of NLE on EQ-5D index value			
**Direct effect**			
NLE→SC	-0.121	-0.140	-0.103
SC→EQ-5D index value	0.122	0.105	0.140
NLE→EQ-5D index value	-0.106	-0.122	-0.090
**Indirect effect**			
NLE→SC→EQ-5D index value	-0.015	-0.018	-0.012
The effect of NLE on EQ-VAS score			
**Direct effect**			
NLE→SC	-0.121	-0.140	-0.103
SC→EQ-VAS score	0.144	0.127	0.161
NLE→EQ-VAS score	-0.105	-0.119	-0.092
**Indirect effect**			
NLE→SC→EQ-VAS score	-0.017	-0.021	-0.014

Note: NLE, negative life events; SC, social capital

Table S13 Direct effects, indirect effects, and the corresponding 95% confidence intervals between the study variables

Model pathways	Standardized coefficient	95% CI	
		Lower bounds	Upper bounds
The effect of NLE on EQ-5D index value			
**Direct effect**			
NLE→SC	-0.105	-0.125	-0.087
SC→EQ-5D index value	0.128	0.110	0.146
NLE→EQ-5D index value	-0.064	-0.082	-0.047
**Indirect effect**			
NLE→SC→EQ-5D index value	-0.013	-0.017	-0.011
The effect of NLE on EQ-VAS score			
**Direct effect**			
NLE→SC	-0.105	-0.125	-0.087
SC→EQ-VAS score	0.148	0.131	0.164
NLE→EQ-VAS score	-0.077	-0.093	-0.063
**Indirect effect**			
NLE→SC→EQ-VAS score	-0.016	-0.019	-0.013

Note: NLE, negative life events; SC, social capital

Table S14 Direct effects, indirect effects, and the corresponding 95% confidence intervals between the study variables

Model pathways	Standardized coefficient	95% CI	
		Lower bounds	Upper bounds
The effect of NLE on EQ-5D index value			
**Direct effect**			
NLE→SC	-0.086	-0.104	-0.068
SC→EQ-5D index value	0.130	0.112	0.148
NLE→EQ-5D index value	-0.060	-0.077	-0.043
**Indirect effect**			
NLE→SC→EQ-5D index value	-0.011	-0.014	-0.009
The effect of NLE on EQ-VAS score			
**Direct effect**			
NLE→SC	-0.086	-0.104	-0.068
SC→EQ-VAS score	0.152	0.135	0.168
NLE→EQ-VAS score	-0.059	-0.073	-0.045
**Indirect effect**			
NLE→SC→EQ-VAS score	-0.013	-0.016	-0.010

Note: NLE, negative life events; SC, social capital
